# Pulmonary Hypertension due to Lung Diseases and/or Hypoxia: What Do We Actually Know?

**DOI:** 10.1155/2017/9598089

**Published:** 2017-08-28

**Authors:** Djuro Kosanovic, Emilio A. Herrera, Akylbek Sydykov, Stylianos E. Orfanos, Elie El Agha

**Affiliations:** ^1^Justus-Liebig University, Giessen, Germany; ^2^University of Chile, Santiago, Chile; ^3^National and Kapodistrian University of Athens, Athens, Greece

Pulmonary hypertension (PH) is a complex, progressive, and life-threatening disease, characterized by increased blood pressure in the lung vasculature. Group III PH includes PH cases that are associated with underlying lung pathology and/or hypoxia, like chronic obstructive pulmonary disease, interstitial lung disease (ILD), obstructive sleep apnea (OSA), alveolar hypoventilation disorders, chronic exposure to high-altitude environment, and developmental lung abnormalities, among others [1, 2].

Current therapy for Group III PH patients aims at treating the underlying cause of lung disease while halting the remodeling process in the pulmonary circulation may offer additional treatments. However, lung transplantation remains the last resort for treating PH in these patients. Despite the clear progress that has been achieved in the past, many clinical and scientific questions still remain not fully determined, described, and understood. Therefore, this special issue covers a wide area of research and presents novel data as well as review articles regarding pulmonary vascular disease which appears due to lung disorders and/or hypoxia.

In this special issue, S. D. Collum et al. provide a timely review about PH associated with idiopathic pulmonary fibrosis and discuss the animal models used to phenocopy and study the human condition. Since pulmonary vascular disease is an additional pathological burden to already severe disease as lung fibrosis [3], the comprehensive and updated information in this article is of great importance to the field. Although it is a well-known fact that high-altitude environment affects the pulmonary circulation, J. Grimminger and colleagues shed light on recently identified mechanisms underlying acute and chronic acclimatization/adaptation and maladaptation to this extreme geographical setting, treatment options, and unresolved issues. They also summarize in vitro, in vivo, and human-based literature regarding hypoxia-induced PH and possible treatment strategies. The consequences of sleep-disordered breathing on right heart structure and function are still unexplored; therefore, A. Maripov et al. review online databases and perform meta-analysis on 25 studies conducted on 1503 OSA patients and 796 healthy subjects. They demonstrate, for the first time, that OSA is associated with right ventricular (RV) wall thickening and dilation as well as dysregulated RV function. N. K. Kozij and coauthors explore feasibility, discriminative validity, and methods of measurement and magnitude of alterations of alveolar and conducting airway-exhaled nitric oxide among healthy human subjects and patients. The study covers many lung diseases and disease subsets such as idiopathic pulmonary arterial hypertension (PAH), ILD, systemic sclerosis (SSc), SSc-associated PAH and ILD, and systemic lupus erythematosus- (SLE-) associated PAH. Last but not least, E. C. C. Castro et al. investigate the expression pattern of serotonin transporter (SERT) during pre- and postnatal periods of human lung development and demonstrate that SERT expression is confined to the endothelium and starts at week 30 of gestation. Interestingly, they also show that alveolar capillary dysplasia with misalignment of pulmonary veins (ACD/MPV) autopsies exhibit diminished SERT expression compared to other neonatal and childhood PH-associated diseases such as bronchopulmonary dysplasia and congenital alveolar dysplasia. ACD/MPV is a rare, yet fatal, developmental lung disorder that affects newborns and infants [4]. Given the difficulty in diagnosing ACD/MPV, testing for the absence of SERT expression postmortem may provide a valuable option for diagnosing this rare disease.

In conclusion, this special issue about pulmonary hypertension due to lung diseases and/or hypoxia reviews the current knowledge about pathophysiological mechanisms and potential treatments but as well highlights the need of more studies in the field to really understand and be able to prevent, early diagnose, and adequately treat PH.

*Djuro Kosanovic* *Emilio A. Herrera* *Akylbek Sydykov* *Stylianos E. Orfanos* *Elie El Agha*
